# Inconsistencies in Neanderthal Genomic DNA Sequences

**DOI:** 10.1371/journal.pgen.0030175

**Published:** 2007-10-12

**Authors:** Jeffrey D Wall, Sung K Kim

**Affiliations:** Institute for Human Genetics, University of California San Francisco, San Francisco, California, United States of America; University of Oxford, United Kingdom

## Abstract

Two recently published papers describe nuclear DNA sequences that were obtained from the same Neanderthal fossil. Our reanalyses of the data from these studies show that they are not consistent with each other and point to serious problems with the data quality in one of the studies, possibly due to modern human DNA contaminants and/or a high rate of sequencing errors.

## Author Summary

One of the enduring questions in human evolution is the relationship of fossil groups, such as Neanderthals, with people alive today. Were Neanderthals direct ancestors of contemporary humans or an evolutionary side branch that eventually died out? Two recent papers describing the sequencing of Neanderthal nuclear DNA from fossil bone held promise for finally answering this question. However, the two studies came to very different conclusions regarding the ancestral role of Neanderthals. In this paper, we reanalyzed the data from the two original studies. We found that the two studies are inconsistent with each other, which implies that the data from at least one of the studies is probably incorrect. The likely culprit is contamination with modern human DNA, which we believe compromised the findings of one of the original Neanderthal DNA studies.

## Introduction

The simultaneous publication of two studies with Neanderthal nuclear DNA sequences [1,2] was a technological breakthrough that held promise for answering a longstanding question in human evolution: Did “archaic” groups of humans, such as Neanderthals, make any substantial contribution to the extant human gene pool? The conclusions of the two studies, however, were puzzling and possibly contradictory. Noonan and colleagues [1] estimated an older divergence time (i.e., time to the most recent common ancestor) between human and Neanderthal sequences (∼706,000 y ago), and a 0% contribution of Neanderthal DNA (95% confidence interval [CI]: 0%–20 %) to the modern European gene pool. In contrast, the Green et al. [2] study found a much more recent divergence time and made two striking observations that were highly suggestive of a substantial amount of admixture between Neanderthals and modern humans.

Specifically, Green et al. [2] reported a human–Neanderthal DNA sequence divergence time of ∼516,000 y (95% CI: 465–569 thousand y) and a human–human DNA sequence divergence time of ∼459,000 y (95% CI: 419–498 thousand y). These two CIs overlap substantially, suggesting that the Neanderthal–human divergence might be within the realm of modern human genetic variation. Indeed, the level of divergence between the Neanderthal sequence (which is primarily noncoding) and the human reference sequence is roughly the same as the average sequence divergence between modern human ethnic groups in noncoding regions of the genome ([3]; J. D. W. and Michael F. Hammer, unpublished results). The second surprising observation from the Green et al. [2] study was that the Neanderthal sequence carries the derived allele for roughly 30% of human SNPs in the public domain [4,5]. For HapMap SNPs [4] alone, the Neanderthal sequence has the derived allele 33% of the time (95% CI: 29%–36 %) while the human reference sequence has the derived allele 38% of the time (95% CI: 34%–42 %). Once again, the overlapping CIs suggest either that humans and Neanderthals diverged very recently—much more recently than is consistent with the fossil record—or that there was a substantial amount of admixture between the two groups. In contrast to the Green et al. study, the comparable percentage from the Noonan et al. [1] data is only 3% (95% CI: 0%–9%).

Noonan et al. [1] and Green et al. [2] used different techniques for sequencing Neanderthal nuclear DNA and estimating parameter values. However, since both studies used genetic material from the same sample, they should come to similar conclusions, even if the particular regions sequenced do not overlap (taking into account the inherent uncertainty in parameter estimation).

To examine the potential discrepancies more carefully, we reanalyzed both the Noonan et al. and Green et al. datasets using a uniform set of methods. We estimated the average human–Neanderthal DNA sequence divergence time, the modern European–Neanderthal population split time, and the Neanderthal contribution to modern European ancestry using methodology very similar to what was used by Noonan et al. [1]. This method is based on a simple population model of isolation followed by instantaneous admixture (see Materials and Methods), and utilizes information from each base pair about whether the human reference sequence and/or the Neanderthal sequence have the derived (or ancestral) allele to estimate parameters. See Noonan et al. [1] for further details.

## Results

To the extent possible, we tried to employ the same data filtering criteria that were used in the original Noonan et al. [1] and Green et al. [2] studies. In total, we analyzed 36,490 base pairs of autosomal Neanderthal sequence from the Noonan et al. [1] study and 750,694 base pairs of autosomal Neanderthal sequence from the Green et al. [2] study. This consists of 100% (Noonan) and roughly 99.8% (Green) of the base pairs analyzed in the two original studies.

We performed analyses similar to Noonan et al. [1] on both datasets (see Materials and Methods). Results that are directly comparable to Figure 5 in Noonan et al. [1] are shown in Figure 1. We describe these results in greater detail below.

**Figure 1:**
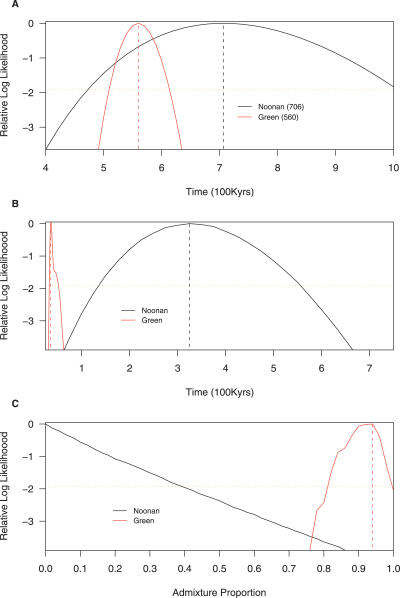
Likelihood Curves for (A) Human–Neanderthal Divergence Time, (B) Modern European–Neanderthal Split Time, and (C) Neanderthal Contribution to Modern European Ancestry for the Noonan et al. (1) and Green et al. (2) Data See Materials and Methods for details.

### Human–Neanderthal DNA Sequence Divergence Time

As in both Noonan et al. [1] and Green et al. [2], we only use human-specific mutations to calculate sequence divergence times. Neanderthal-specific mutations are excluded because the vast majority of these (∼90%) are thought to be caused by post-mortem DNA damage [1,2]. We estimate sequence divergence times of 706 thousand y ago [Kya] (95% CI: 466–1,028 Kya) for the Noonan et al. [1] data and 560,000 Kya (95% CI: 509–615 Kya) for the Green et al. [2] data. For comparison, the estimates from the original studies were 706 Kya (95% CI: 468–1,015 Kya) and 516 Kya (95% CI: 465–569 Kya), respectively. The slight discrepancies between our estimates and the Green et al. [2] and Noonan et al. [1] ones arise due to minor methodological differences in sequence divergence time estimates between the two original studies and to slight differences in the way that CIs were calculated (see Materials and Methods).

### Modern European–Neanderthal Population Split Time

Our estimates of population split times differ markedly between the two datasets. We estimate a modern European-Neanderthal population split time of 35 Kya (95% CI: 33–51 Kya) for the Green et al. data and a split time of 325 Kya (95% CI: 135–557 Kya) for the Noonan et al. data. Figure 1B shows that these two estimates are clearly not consistent with each other. The original Noonan et al. study used slightly different methodology to estimate an even older split time (440 Kya; 95% CI: 170–620 Kya), while the Green et al. study did not estimate population split times from their data. Note that for the model used here the population split time is always much more recent than the sequence divergence time (see Materials and Methods).

### Neanderthal Contribution to Modern European Ancestry

The estimates of the Neanderthal contribution to modern European ancestry are also dramatically discordant between the two datasets. When we fix the modern European–Neanderthal split time at 325 Kya (as estimated from the Noonan et al. data), the Neanderthal admixture estimates are 94% (95% CI: 81%–100 %) for the Green et al. data and 0% (95% CI: 0%–39 %) for the Noonan et al. data. These admixture estimates are dependent on the assumed modern European–Neanderthal population split time. Only if we arbitrarily fix a very recent split time of 60 Kya or less are the admixture estimates from the two datasets compatible with each other (unpublished data). However, this very recent split time is not consistent with the estimated modern European–Neanderthal population split time from the Noonan et al. data [1] (Figure 1B).

## Discussion

Given the large discrepancies in the parameter estimates from the two studies, it is clear that the conclusions reached by at least one of the studies are incorrect. We examine the two possibilities in greater detail below.

If the estimates from the much larger Green et al. [2] dataset are accurate, then the discrepancies in Figures 1B and 1C either arose by chance (i.e., due to the uncertainty in the estimates from the much smaller Noonan et al. dataset) or due to some unknown bias or problem with the Noonan et al data. Under this scenario, either the modern European–Neanderthal split time is very recent (i.e., ≤60 Kya) or the Neanderthal admixture proportion is extremely high (>>50%). Paleoanthropological evidence suggests that Neanderthals formed a distinct group of fossils at least 250 Kya [6,7], so the more recent modern European–Neanderthal split time is highly unlikely. In addition, most of the available evidence from paleoanthropology suggests that the Neanderthal contribution to the modern gene pool is limited [7], while previous Neanderthal mtDNA studies concluded that the Neanderthal contribution could be no more than 25% [8,9]. Furthermore, preliminary analyses of additional nuclear Neanderthal sequences suggest a much older human–Neanderthal sequence divergence time than was found by Green et al. [10]. So, based on studies from the same [9,10] and results from other laboratories [6–8], it seems extremely improbable that the Green et al. estimates are accurate.

In contrast, if the Noonan et al. [1] estimates were correct, then no additional assumptions would be needed to understand the Neanderthal nuclear DNA sequence data in the context of previous human evolutionary studies. This leads to consideration of three possible issues that may have compromised data quality in the Green et al. [2] study: contamination with modern human DNA, widespread difficulties in aligning Neanderthal DNA fragments, and abnormally high DNA sequencing error rates.

Although Green et al. [2] found little evidence of modern human mtDNA contamination, it is not clear whether this observation generalizes to the autosomal data under study. To examine this in greater detail, we divided the Green et al. sequence data into three groups: short (≥30 bp and ≤50 bp) fragments, medium-sized (>50 bp and ≤100 bp) fragments, and large (>100 bp) fragments (see Materials and Methods). We then estimated the human–Neanderthal sequence divergence time for each of these groups. The likelihood of the data as a function of the divergence time is shown in Figure 2. While the short fragments have an estimated divergence time similar to what was found in the Noonan et al. [1] study, the large fragments are much more similar on average to modern human DNA. In fact, the large fragments have an estimated human–Neanderthal sequence divergence time that is *less* than the estimated divergence time between two Hausa (West African) sequences (see Materials and Methods). If true, this would indicate greater similarity between human and Neanderthal than between two extant members of the Hausa population. This pattern thus raises the concern that some of the longer sequence fragments are actually modern human contaminants. Modern human contamination would be expected to be size biased, since actual Neanderthal DNA would tend to be degraded into short fragments [1,11]. We note that the observation of a length dependence of the results makes alignment issues alone [10] unlikely to be a sufficient explanation, since we would expect that longer fragments would be easier to align and thus the data from longer fragments should be more accurate. We further note that we did not find a similar signal of potential contamination in the Noonan et al. data (unpublished data).

**Figure 2:**
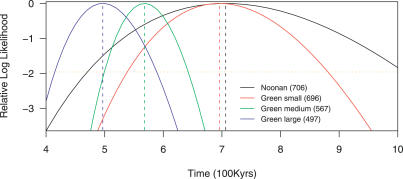
Relative Likelihood Curve for the Human–Neanderthal Divergence Time The Green et al. [2] data are divided into short (≤50 bp), medium (>50 and ≤100 bp), and large (>100 bp) fragments.

We also tabulated the percentage of HapMap SNPs for which the Neanderthal sequence contains the derived allele for each of the three groups of Green et al. data (see Table 1), and refer to this percentage as *N_d_*. For comparison, we also estimated (from simulations) the expected value of *N_d_* as a function of the European–Neanderthal population split time and the Neanderthal admixture proportion (see Figure 3). The expected value of *N_d_* increases as the European–Neanderthal population split time decreases and/or the Neanderthal admixture proportion increases, though for reasonable parameter values (i.e., a population split time ≥150 Kya and a Neanderthal admixture proportion <25%) *N_d_* is always <25%. In contrast, *N_d_* = 32.9 for the Green et al. data, which is inconsistent with the true value of *N_d_* being ≤25% (*p* < 10^−4^). Moreover, *N_d_* shows a clear trend of higher values for longer fragments, consistent with the hypothesis of widespread contamination with larger-sized modern human DNA fragments. The expected value of *N_d_* for modern human contaminants is 37.0, so the Green et al. data look in some ways more like modern human DNA than they do like Neanderthal DNA. If we use *N_d_* to estimate very roughly the proportion of the Green et al. [2] data that are actually modern human contaminants, this leads to a contamination rate estimate of 73% (95% CI: 51%–97%; see Materials and Methods). Alternatively, a likelihood-based estimate of the proportion of modern human contaminants yields an estimate of 78% (95% CI: 70%–88%; see Materials and Methods). These estimates are very approximate, and given the uncertainty in the actual *N_d_* values for Neanderthal and modern human DNA, the data are also consistent with lower (but still substantial) levels of contamination.

**Table 1:**
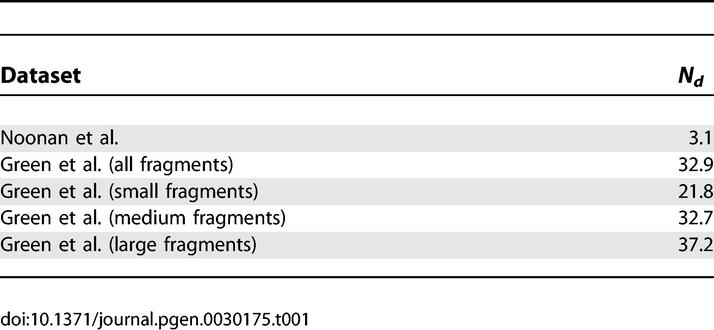
*N_d_* Values for the Noonan et al. [1] and Green et al. [2] Datasets

**Figure 3:**
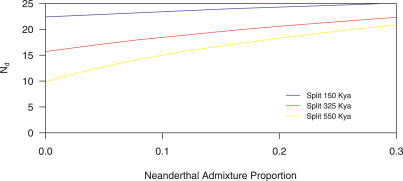
Estimates of *N_d_* as a Function of the Neanderthal Admixture Proportion and the Modern European–Neanderthal Population Split Time

On the other hand, if contamination were prevalent in the Green et al. [2] data, then due to the large number of apparent mutations that appear in Neanderthal DNA due to post-mortem DNA damage [1,2] the Neanderthal-specific sequence divergence should be smaller for the Green et al. data than it is for the Noonan et al. data. Instead, we find that the Neanderthal-specific divergences are almost the same for the two studies. It is not clear how to interpret this observation. It is clear that the two studies are inconsistent with each other, and given the weight of evidence from many previous studies, the more parsimonious explanation is still that something is wrong with the Green et al. [2] data. Although we have highlighted strong circumstantial evidence of modern human DNA contamination in the Green et al. data, there are likely other important problems or biases affecting data quality in one or both studies. One possibility is that due to subtle differences in laboratory protocols, there is a higher sequencing error rate in the Green et al. data than in the Noonan et al. data. (Sequencing errors would look the same as Neanderthal-specific mutations in our analyses.) There is some indirect evidence of this—post-mortem DNA damage often causes the deamination of cytosine to uracil [12], resulting in apparent C→T or G→A mutations. These particular mutations make up a significantly larger fraction of the Neanderthal-specific mutations from the Noonan et al. data than they do for the Green et al. data (χ^2^, 1 degree of freedom; X^2^ = 21.2, *p* << 10^−4^), suggesting that some other process (besides post-mortem damage to Neanderthal DNA) is leading to the “Neanderthal-specific mutations” in the Green et al. data.

In conclusion, the sequencing of Neanderthal nuclear DNA is truly a remarkable technical achievement. However, because contamination with modern human DNA and sequencing error rates are continuing concerns, it will be important to carefully evaluate published and future data before arriving at any firm conclusions about human evolution.

## Materials and Methods

### Data.

Neanderthal sequence data was obtained from the supplementary online material in Noonan et al. [1] and Green et al. [2]. The mapping coordinates of the Green et al. [2] alignments of the Neanderthal, reference human, and chimpanzee sequences are based on National Center for Biotechnology Information (NCBI) build 36 of the human genome. We removed 5 bp of sequence from the ends of each read, and analyzed fragments that contained at least 30 bp of autosomal sequence. When multiple sequence fragments overlapped with each other, we analyzed the largest one and threw out the other(s). For the Noonan et al. [1] study, all of the relevant information was directly available from their Supplementary Methods. For the Green et al. [2] study, we did not have access to their post-filtered dataset for comparison. Using the above criteria, we started with 891,406 total bases from 13,955 unique fragments. Of these, we removed 115,963 bases with missing data or a poor-quality score, 24,698 bases found on the sex chromosomes, and 51 bases where the human, Neanderthal, and chimpanzee sequences all had different bases, leaving 750,694 bp for our analyses. Green et al. [2] do not specify the total number of base pairs used in their analyses, but they do mention that 736,941 base pairs are identical across human, Neanderthal, and chimpanzee. Our filtering criteria yielded 735,878 such base pairs, within 0.2% of what was analyzed in Green et al. [2].

For all biallelic HapMap SNPs (available in March 2007) that overlap with the Neanderthal autosomal sequences, we used a human–chimpanzee alignment to determine which allele was derived or ancestral. This alignment is available at http://hgdownload.cse.ucsc.edu/goldenPath/hg18/vsPanTro1/, and uses NCBI build 36 of the human genome and the PanTro1 build of the chimpanzee genome. HapMap SNPs without an orthologous chimpanzee reference allele were excluded, as were SNPs that did not segregate in the Utah residents with ancestry from northern and western Europe (CEU) population. CIs for the proportion of HapMap SNPs where the Neanderthal (or human reference) sequence had the derived allele were estimated from 5,000 bootstrap simulations.

### Sequence divergence time estimates.

When comparing Neanderthal–modern human divergence with human–human divergence, we ignored Neanderthal-specific mutations, since most of them are due to post-mortem DNA damage [1,2]. Using the divergence on the human-specific branch to estimate sequence divergence times is expected to be unbiased. For comparison, π for two Hausa individuals is roughly 0.11% [3], and is roughly 0.12% for two individuals from other African populations (JDW, unpublished results). These π values are for noncoding DNA and should be compared with twice the divergences displayed in Table 1. Over 90% of the Neanderthal base pairs from the original studies map to noncoding (putatively nonfunctional) areas of the human genome.

### Model and data analysis.

We follow the model outlined in Noonan et al. [1] for the CEU population. A schematic of the model is shown in Figure 4 (see also Figures S3 and S6 from Noonan et al.). The parameters estimated are *T*_MRCA_, the average human–Neanderthal DNA sequence divergence time; *T*_s_, the modern European–Neanderthal population split time; and *p*, the Neanderthal contribution to modern European ancestry. From Figure 4, it is clear that *T*_MRCA_ is always substantially larger than *T*_s_. Estimates of *T*_s_ assume that *p* = 0, while estimates of *p* assume that *T*_s_ = 325 Kya. See Noonan et al. [1] for further details.

**Figure 4:**
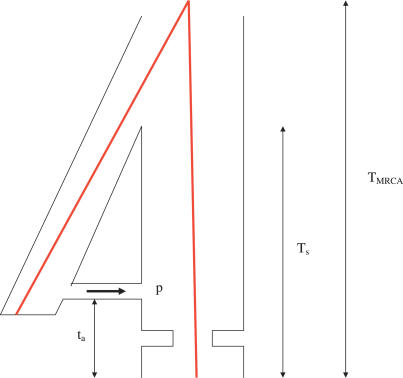
Schematic of the Population Model Used The Neanderthals contribute a proportion *p* to the ancestry of CEU individuals *t*_a_ generations in the past, where *t*_a_ = 1,800 (see Noonan et al. [1]). *T*_s_ is the split time of the two populations. The red lines show the ancestry of a particular Neanderthal and modern human sequence. They coalesce at time *T*_MRCA_ in the past.

Our analyses are similar to those described in Noonan et al. [1], but with several minor differences. For HapMap SNPs we categorize them into ten different frequency bins when calculating *P* (Ascert|freq) and when tabulating ascertained SNPs by frequency (see page 10 of Supplementary Online Material from [1]). These bins consist of frequencies (0.0, 0.1], (0.1, 0.2], . . . (0.9, 1.0). Summaries of the actual data analyzed are provided in Tables 2 and 3. The parameter values and demographic models used were the same as in [1], except we took a mutation rate of 2.5 × 10^−8^/bp.

**Table 2:**
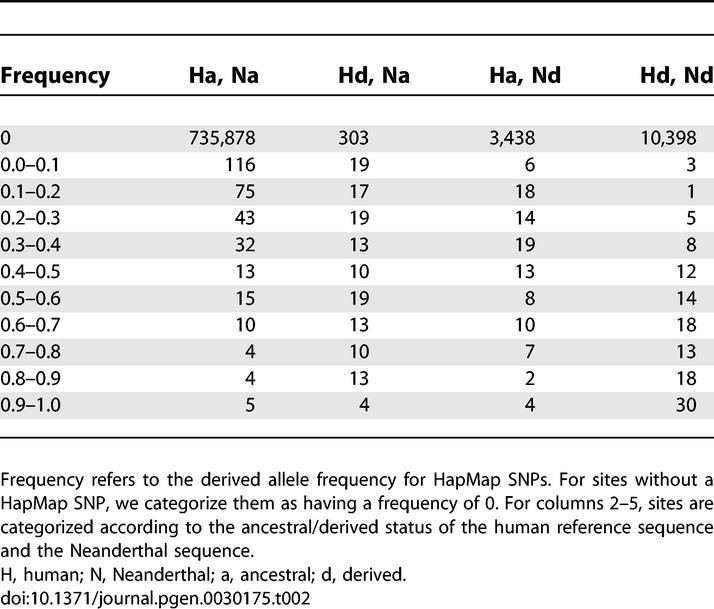
Summary of Data Analyzed from Green et al. [2]

**Table 3:**
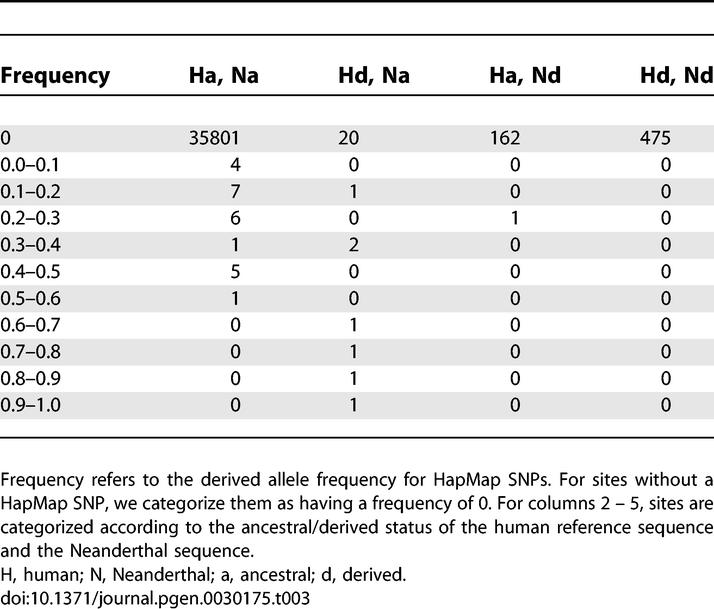
Summary of Data Analyzed from Noonan et al. [1]

For estimating split times, we ran simulations for split times in 5,000-y increments (for times less than or equal to 50 Kya) or 25,000-y increments (for split times greater than 50 Kya). For estimating Neanderthal admixture, we fixed the population divergence time at 325 Kya and ran simulations in increments of 0.02 (i.e., 0, 0.02, 0.04, etc.) for the admixture proportion. Results are based on 50 million replicates for each set of parameter values. Approximate 95% CIs were estimated assuming the composite likelihood was a true likelihood (as well as the standard asymptotic assumptions for maximum likelihood). Since each fragment is so small, assuming each site is independent is a reasonable approximation.

### Contamination estimates.

We assume that the true Neanderthal admixture rate is 0 (as estimated from the Noonan et al. data), that the true modern European–Neanderthal population split time is 325 Kya (as estimated from the Noonan et al. data), and that the Green et al. data consist of a simple mixture of true Neanderthal DNA (with *N_d_* = 21.8, cf. Table 1, Green small fragments) and modern human DNA (with *N_d_* = 37.0). We estimate contamination rates in two ways. First, we find the proportion of Neanderthal versus modern human DNA that gives an expected *N_d_* value equal to 32.9 (the actual value in the Green et al. data). Second, we find what proportion of Neanderthal versus modern human DNA maximizes the (composite) likelihood described above. We note that the likelihood of the data for a model with 78% contamination is modestly higher (1.06 log-likelihood units) than the likelihood of the data for a model with no contamination and 94% admixture (maximum-likelihood in Figure 1B).

## Abbreviations

CEU: Utah residents with ancestry from northern and western Europe
CI: confidence interval
Kya: thousand y ago
